# Deep brain stimulation of the subthalamic nucleus improves sleep in Parkinson disease patients: A systematic review and meta-analysis

**DOI:** 10.1097/MD.0000000000034509

**Published:** 2023-08-11

**Authors:** Keying Zhu, Sun Peng, Yulun Wu, Yuanyuan Zhao, Zhonglei Lu

**Affiliations:** a Center for Rehabilitation Medicine, Rehabilitation and Sports Medicine Research Institute of Zhejiang Province, Department of Rehabilitation Medicine, Zhejiang Provincial People’s Hospital, Affiliated People’s Hospital, Hangzhou Medical College, Hangzhou, Zhejiang, China; b Department of Neurosurgery, Zhejiang Provincial People’s Hospital, Affiliated People’s Hospital, Hangzhou Medical College, Hangzhou, Zhejiang, China.

**Keywords:** deep brain stimulation, meta-analysis, Parkinson disease, sleep quality, subthalamic nucleus

## Abstract

Sleep disorders significantly affect the quality of life in Parkinson disease (PD) patients. Deep brain stimulation of the subthalamic nucleus has been reported to improve motor symptoms and decrease medication usage. However, the impact of subthalamic nucleus deep brain stimulation (STN-DBS) on sleep quality in PD patients remains to be definitively determined. This systematic review and meta-analysis, conducted following the preferred reporting items for systematic reviews and meta-analyses guidelines, aimed to clarify the effect of STN-DBS on sleep quality in PD patients.

**Methods:**

A rigorous literature search identified 6 studies, including 1 randomized controlled trial and 5 self-controlled trials, totaling 154 patients who underwent deep brain stimulation, providing 308 pairs of data for analysis. Parkinson disease sleep scale was the primary measure of interest, while the Movement Disorder Society-sponsored revision of the unified Parkinson disease rating scale was documented in all trials. Study quality was assessed using the Newcastle-Ottawa scale.

**Results:**

STN-DBS significantly improved Parkinson disease sleep scale scores (mean difference = 20.41, 95% CI: [13.03, 27.79], *I*² = 60.8%, *P* < .001), indicating enhanced sleep quality. Furthermore, a significant reduction in movement disorder society unified Parkinson disease rating scale part III scores postoperatively (mean difference = −12.59, 95% CI: [−14.70, −10.49], *I*² = 89.9%, *P* < .001) suggested improved motor function. PD medication usage was also significantly reduced postoperatively (mean difference = −314.71, 95% CI: [−468.13, −161.28], *I*² = 52.9%, *P* < .001). A sensitivity analysis confirmed the robustness of the main findings. The sample size was adequate, allowing for conclusive inferences.

**Conclusion:**

The present study, which comprises a comprehensive systematic review and meta-analysis, offers compelling evidence that STN-DBS can ameliorate sleep quality, augment motor function, and curtail medication consumption among individuals afflicted with PD.

## 1. Introduction

Parkinson disease (PD) is the second most common neurodegenerative disorder, surpassed by Alzheimer disease. However, the exact cause of Parkinson remains unclear. Parkinson disease may arise due to a variety of factors, including, but not limited to, aging, heredity, environmental influences, and additional factors. The manifestation of Parkinson in patients is characterized by the presence of pathological alterations in the formation of Lewy bodies as well as the gradual degeneration of dopaminergic neurons located in the substantia nigra. This may lead to a reduction in the secretion of dopamine neurotransmitters in the striatum, and an imbalance in the levels of dopaminergic and acetylcholinergic neurotransmitters. The cardinal motor manifestations of Parkinson include limb tremor, muscular stiffness, bradykinesia, and postural instability. A plethora of non-motor symptoms are associated with Parkinson, including but not limited to sensory disorders, sleep disorders, autonomic nervous dysfunction, behavioral disorders, and depression.

Recently, there has been a growing trend among patients to opt for surgical interventions as a means of mitigating the motor symptoms of Parkinson disease, owing to continued research and investigation into the pathological mechanisms and treatment options for this condition. The efficacy of deep brain stimulation (DBS) in mitigating the tardive motor symptoms of Parkinson is well established. However, the impact of DBS on non-motor symptoms of Parkinson remains a topic of debate. Several studies have indicated that DBS surgery may induce or aggravate behavioral disorders among patients, whereas others have shown that DBS surgery can mitigate specific mental symptoms among patients.^[1]^ According to research findings, Parkinson may present with sleep disturbances as its initial clinical manifestation.^[2]^ The prevalence of sleep disorders in Parkinson disease varies between 40% and 98 percent,^[3]^ thereby exerting a substantial influence on patients quality of life and disease prognosis. The precise etiology of sleep disturbances in individuals with Parkinson remains unclear. It is postulated that the degeneration of the brainstem sleep-awakening center and thalamic cortex pathway may cause or intensify sleep disorders. Several animal studies have indicated that sleep disturbances and heightened arousal associated with Parkinson are a result of their toxic impact on dopaminergic neurons. Furthermore, the impact of motor symptoms, pharmacological interventions, and nonpharmacological therapies on sleep patterns in individuals with Parkinson disease and affective disorders exhibit diverse outcomes.

DBS is a novel therapeutic approach used to ameliorate symptoms in patients and address functional brain disorders. This method involves the implantation of minute electrodes into precise locations on the patient’s brain through stereotactic surgery. Subsequently, high-frequency low-intensity electrical pulses were emitted through the electrodes to stimulate the relevant nuclei. Theoretically, DBS does not cause permanent damage to brain tissue. At present, it is frequently utilized for the management of dystonic conditions, including Parkinson disease, epilepsy, essential tremor, and torsion dystonia. DBS has been shown to achieve optimal symptom management with minimal side effects via postoperative programming. Furthermore, DBS is characterized by its definitive, safe, and reversible effects, distinguishing it from previous nuclear destruction procedures. A growing number of patients are electing to undergo surgical intervention to mitigate the motor symptoms associated with Parkinson disease. As a result, there has been notable improvement in these symptoms.^[4]^ Hence, it is crucial to understand the impact of DBS on sleep patterns in individuals with PD.

The Parkinson disease sleep scale (PDSS), which was recently developed, provides insight into the subjective impact of DBS on sleep quality and nocturnal disability.^[5]^ The PDSS is a self-assessment tool comprising 15 items scored based on the degree of visual stimulation, with a scale ranging from 0 (indicating a consistently severe presence) to 10 (indicating an absence of symptoms). Upon summing these scores, it can be inferred that a higher score corresponds to a superior quality of sleep.

In this case, a subsequent meta-analysis was performed to evaluate the influence of enhanced sleep on the utilization of Subthalamic Nucleus Deep Brain Stimulation (STN-DBS) in individuals with Parkinson disease.

## 2. Materials and methods

A systematic review was carried out with a rigorous approach, adhering to the established guidelines outlined by the preferred reporting items for systematic reviews and meta-analyses.^[6]^ The results are presented in accordance with these guidelines. The manuscript in question did not require ethical approval or informed consent, as all data were obtained from preexisting, published sources. A pair of investigators independently carried out the study retrieval, eligibility determination, data extraction, and quality evaluation. The researchers engaged in collaborative discourse to address the discrepancies encountered, which required them to reach mutual agreement.

### 2.1. Selection of studies

We thoroughly searched PubMed, Embase, Web of Science, and Cochrane Library in order to ensure a thorough search across all 4 electronic databases. There were no temporal constraints placed on the search, which was conducted from each database’s creation until May 6, 2023. According to each database’s unique requirements, the vocabulary and syntax were changed. An organized search was done in the PubMed database using terms like “DBS,” “deep brain stimulation,” “Parkinson disease,” “PDSS,” “sleep quality,” and “PD.” This study was conducted in any language. Furthermore, to find any possibly overlooked entries, the reference lists of pertinent papers were manually screened.

### 2.2. Inclusion criteria

Only clinical studies that fulfilled the following requirements were included in this study: Patients with Parkinson disease who had DBS to the subthalamic nucleus (STN) and had pre- and postoperative PDSS scores computed. In this analysis, only original research publications were incorporated. Review articles were only used to locate additional pertinent material. The study did not include case reports.

### 2.3. Data extraction

Literature screening and data extraction were performed by 2 evaluators in a manner that ensured independence. The evaluators cross-verified each other’s work and any discrepancies that arose were discussed and resolved. In the process of literature screening, titles and abstracts were first scrutinized to exclude irrelevant articles. This was followed by a comprehensive evaluation of the full text to ascertain inclusion. The original articles were used to extract patient demographics, including age, sex, religion, disease duration, and relevant study outcomes. The present study presents the results of an analysis conducted on the PDSS, Unified Parkinson Disease Rating Scale III, and medication dosage data collected from patients diagnosed with Parkinson disease. In cases where data were presented as medians and (interquartile) ranges, the authors in charge were contacted to acquire the corresponding means and standard deviations. In instances where the results were exclusively presented in a graphical format, pertinent data were obtained from the original authors. Two authors utilized the Newcastle-Ottawa Scale to evaluate the methodological quality and risk of bias of each included study.

### 2.4. Quality assessment

The Newcastle-Ottawa scale (NOS) was used to appraise the quality of the studies included.^[7]^ The scale is divided into 3 distinct domains: Selection, Comparability, and Outcome/Exposure, comprising a cumulative total of 9 items, with a maximum achievable score of 9 points. The NOS components were evaluated by 2 reviewers, who included the representativeness of the exposed cohort, selection of the non-exposed cohort (selection bias), ascertainment of exposure, demonstration of outcome, comparability of cohorts (comparability bias), evaluation of outcome, sufficiency of follow-up duration, and adequacy of cohort follow-up (outcome bias). Study quality was classified into 3 categories based on the scores falling within the ranges of 0 to 3, 4 to 6, and 7 to 9, which were representative of low, medium, and high study quality, respectively.

### 2.5. Statistical analysis

The assessment of heterogeneity among studies was conducted using chi-square statistics and was defined by the extent of *I*². The *I*² statistic was used to evaluate heterogeneity among the included studies. When the *I*² value was equal to 0%, heterogeneity was not observed. However, if the *I*² value exceeds 50%, it signifies a considerable degree of heterogeneity. Relative risks were standardized within each article and then aggregated using a random effects model. Furthermore, a sensitivity analysis was performed to determine whether the outcomes of the overall study were significantly influenced by the exclusion of a single study using a one study-removed methodology. Funnel plot symmetry and Egger test were used to investigate the presence of publication bias in meta-analyses comprising ten or more eligible articles. In cases where the funnel plot exhibits asymmetry, it is deemed that the assumption that unpublished negative studies contribute to publication bias has a notable impact on the estimation of the effect. Statistical significance was determined in all tests by considering 2-sided *P* values that were less < 0.05. The statistical software STATA version 17 (Stata Corp, College Station, TX) was used to analyze data obtained from randomized controlled trials (RCTs) that met the inclusion criteria.

## 3. Results

### 3.1. Search results

**Figure 1.**
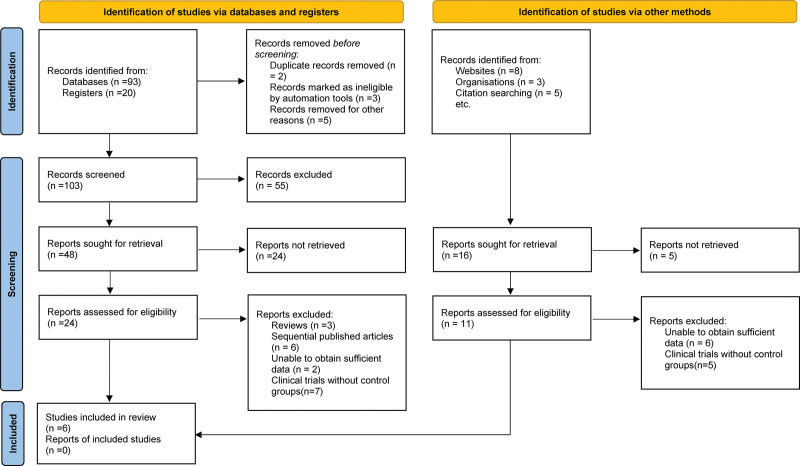
Selection process of included studies.

The literature search yielded 113 articles (Fig. 1). Following the removal of duplicate entries, 108 articles remained. Following a screening process based on titles and abstracts, 18 reviews, 22 animal studies, and 46 unoperated patients were excluded. Subsequently, we examined 22 full text reports. After conducting a comprehensive inquiry, it was determined that eleven reports were excluded due to insufficient PDSS. Three supplementary instances were eliminated because of the absence of an average indigenous standard deviation.^[8–10]^ A study was conducted to evaluate the sleep quality of patients with Parkinson who underwent treatment with globus pallidus internus deep brain stimulation.^[11]^ Subsequently, 2 articles were removed from our core corpus on the possibility of membership in a redundant cluster.^[12,13]^ The previously computed data were subsequently extracted and referred to as Yi2018 in our meta-analysis. As a result, this study selected 6 discrete articles that reported on 154 patients who underwent deep brain stimulation, comprising 308 pairs, for the purpose of analysis. Given the rigorous selection process and the consistency of the results across the studies, we believe that the sample size of 154 patients across 6 studies is adequate for making meaningful inferences in our meta-analysis.

### 3.2. Description of included trials

Tables 1 and 2 summarize the primary features of the inclusion trial. This study included 6 experiments. The study included 6 trials, consisting of one RCT and 5 self-controlled trials. The mean age at onset was approximately 60 years, and the disease duration exceeded 10 years. The experimental population was predominantly composed of males. The range of sample sizes observed in this study was 5 to 63. The study was a RCT, wherein individuals who received STN-DBS were chosen as subjects for analysis.^[14]^ The principal measure of interest in this meta-analysis was the PDSS, whereas the Movement Disorder Society-sponsored revision of the unified Parkinson disease rating scale (MDS-UPDRS-III) was documented in all trials that were incorporated. The study reported 3 trials on drug therapy for Parkinson disease, and the quality of each study was assessed by 2 evaluators using the NOS. All the trials that were incorporated in the study received a rating of 4 stars, and the overall score on the Newcastle-Ottawa Scale was 99. The absence of a control group was the principal justification for the deduction of marks.

**Table 1:** The quality assessment according to NOS of each cohort study.

study	Selection				Comparability	Outcome			Total score
	Representativeness of the exposed cohort	Selection of the non -exposed cohort	Ascertainment of exposure	Demonstration that outcome	Comparability of cohorts	Assessment of outcome	Was follow-up long enough	Adequacy of follow-up of cohorts	
Namiko et al 2011		★	★	★	★★	★	★	★	8
Chahine et al 2011	★	★	★	★	★★	★	★	★	9
Peppe et al 2012	★	★	★	★	★	★	★	★	8
Vincent et al 2013	★	★		★	★	★	★	★	7
Merlino et al 2014	★	★	★	★	★★	★		★	8
Yi et al 2018	★	★	★	★	★	★	★	★	8

NOS = New Castle-Ottawa scale.

**Table 2:** The features of the included investigations.

Author	Year	Age	Country	Sample size	Gender (male/female)	Disease duration	Study outcomes
Nishida Namiko	2011	57.5 ± 9.8	Japan	10	4/6	12.3 ± 2.7	PDSS, PSG, UPDRS-III
LM. Chahine	2011	62 ± 8.8	USA	17	11/6	13.1 ± 8	PDSS, ESS, UPDRS-III, Parkinsonian medication
Antonella Peppe	2012	62.8 ± 1.9	Italy	5	NA	10.2 ± 3.8	PDSS, ESS, UPDRS-III, Parkinsonian medication, MMSE
Vincent JJ odekerken	2013	60.9 ± 7.6	Netherland	63	44/19	12.0 ± 5.3	PDSS, UPDRS-III, ALDS, PDQL, adverse events
Giovanni Merlino	2014	NA	Italy	15	11/4	10.6 ± 3.8	PDSS, PSG, UPDRS-III, sleep latency, TST day sleepiness, RLS
Yi	2018	56.9 ± 6.5	China	88	44/44	11.6 ± 3.6	PDSS, PSG, UPDRS-III, sleep latency, TST day sleepiness, RLS

ESS = Epworth sleepiness scale, PDSS = Parkinson disease sleep scale, RLS = restless legs syndrome, UPDRS = unified Parkinson disease rating scale.

### 3.3. Primary outcome

**Figure 2.**
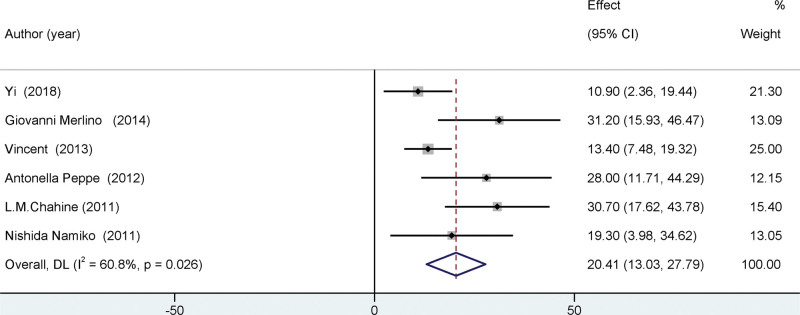
Forest plots of the PDSS. In the pooled results, PDSS increased markedly after surgery. It was found that sleep quality improved after surgery. PDSS = Parkinson disease sleep scale.

Our meta-analysis incorporated 6 studies on PDSS, and the outcomes are depicted in the initial forest plot. (See Fig. 2). DBS was found to have a significant impact on enhancing PDSS scores (mean difference = 20.41, 95% confidence interval = [13.03, 27.79], *I*² = 60.8%, *P* < .001). These findings suggest that DBS has the potential to significantly enhance PDSS scores over an extended duration of monitoring.

**Figure 3.**
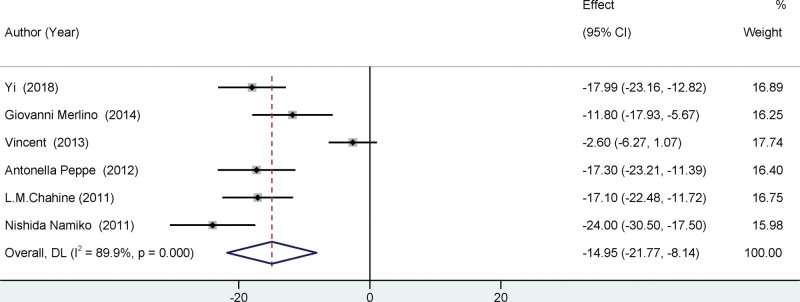
Forest plots of the UPDRS-III. The pooled results indicated a significant improvement in motor function following surgery. UPDRS = unified Parkinson disease rating scale.

**Figure 4.**
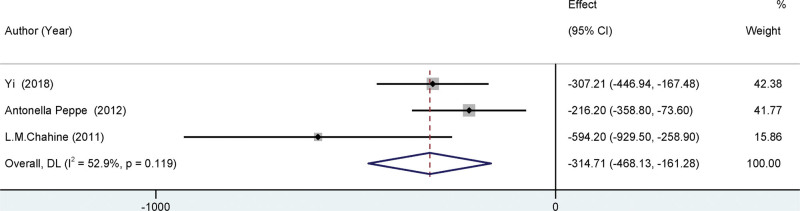
Forest plots of the LEDD. After deep brain stimulation, the pooled results of LEDD revealed a significant decrease in medication use.

All the trials that were incorporated in the present meta-analysis reported the MDS-UPDRS-III, as depicted in Figure 3. The study found a statistically significant difference in MDS-UPDRS-III scores before and after surgery, with a mean difference of −12.59 (95% CI = [−14.70,10.49], *I*²=89.9%, *P* < .001). The findings indicated a significant decrease in all the scores following the surgical procedure. These results suggest a noteworthy enhancement in motor function in individuals who underwent STN-DBS treatment. Three of the studies included in the analysis documented pharmacological interventions for individuals diagnosed with Parkinson. (See Fig. 4). The study found a significant decrease in Parkinson disease medication postoperatively, with a mean difference of −314.71, and a 95% confidence interval ranging from −468.13 to −161.28. The heterogeneity of the results was moderate, with an *I*² value of 52.9%. The *P* value was <.001, indicating statistical significance.

### 3.4. Results of quality assessment

We assessed the methodological quality of each RCT using the NOS. In general, 1 study scored 7 points, 4 studies scored 8 points, and 1 study scored 9 points. No studies were blinded and there was no evidence of allocation concealment. No funding bias was evident in any of the studies. No studies had incomplete outcome data, early stoppage bias, or baseline imbalances. The risks of bias and corresponding ratios are summarized (Table 1).

### 3.5. Sensitivity analysis

**Figure 5.**
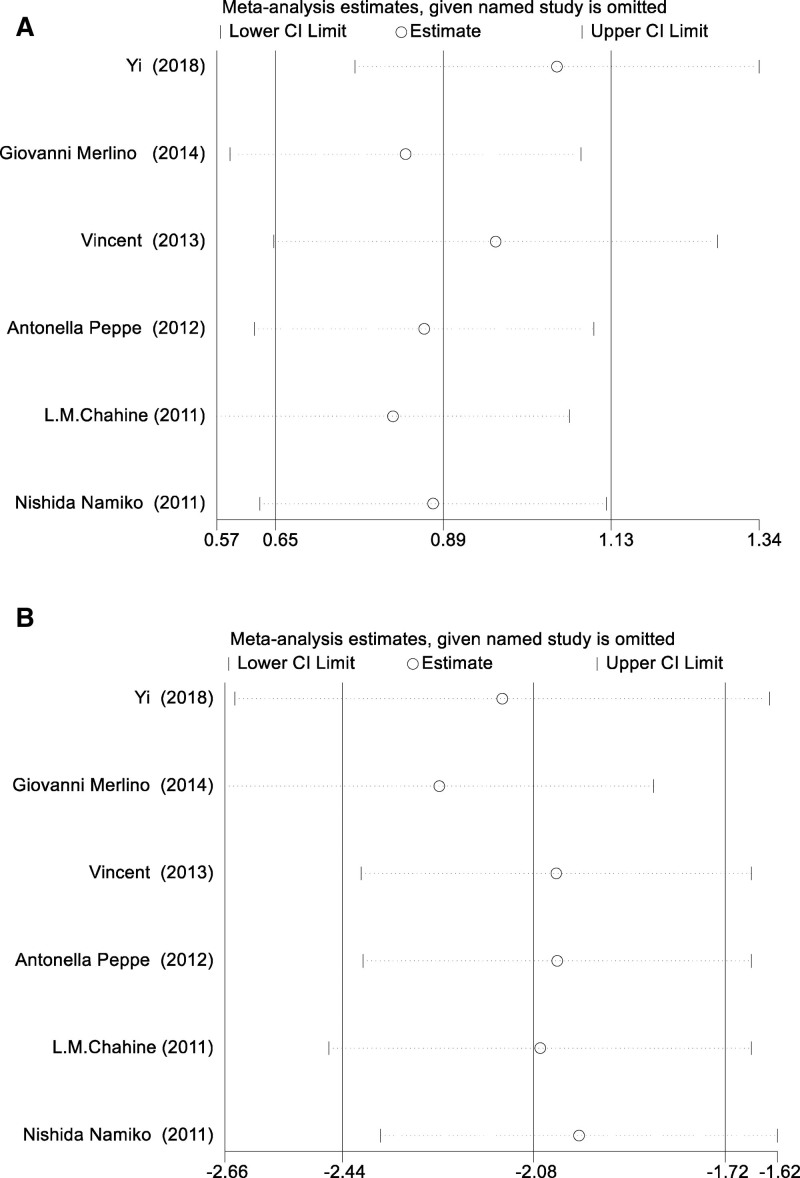
Sensitivity analysis of the PDSS (A) and UPDRS-III (B). PDSS = Parkinson disease sleep scale. UPDRS = unified Parkinson disease rating scale.

A sensitivity analysis was performed to evaluate the stability and dependability of the aggregated outcomes, owing to the significant heterogeneity observed among the studies incorporated in the meta-analysis. The present study conducted an analysis whereby each individual study was sequentially excluded, and the combined effect estimates were recalculated for the remaining studies. The conducted sensitivity analysis was thorough and demonstrated that the combined findings remained consistent and resilient even with the omission of any individual study. This suggests that the outcomes of our research are more dependable as no single study exerted an excessive impact on the overall results. The consistency of the outcomes throughout these analyses highlights the resilience of our principal discoveries and reinforces the inferences derived from this meta-analysis. (Fig. 5).

## 4. Discussion

This Meta-analysis presents a compilation of evidence indicating that STN-DBS has a significant positive impact on sleep quality. Furthermore, the enhancement of motor function after surgery was concomitant with a decrease in medication use for Parkinson disease. No additional tests for self-regulation were required.

Non-motor symptoms of PD, known as sleep disorders, are widely prevalent and have a considerable detrimental impact on the quality of life of patients. Although the sleep disorders experienced by individuals with PD bear resemblance to those of the wider population, unique considerations exist with respect to diagnosis, management, and impact. Patients with Parkinson disease frequently experience insomnia, which can manifest as difficulty initiating sleep, early morning awakenings, or waking up frequently throughout the night. The occurrence of rapid eye movement (REM) sleep behavior disorders can be attributed to the advancement of a pathological condition, unfavorable impacts of medication, or correlated indications such as anxiety or depression. This disorder is characterized by rapid eye movement. The rapid eye movement sleep behavior disorder (RBD) is characterized by anomalous body movements and dream experiences during the REM sleep phase, as documented in reference.^[3]^ On the other hand, restless leg syndrome (RLS) is a neurological ailment that induces discomfort in the legs during rest and an intense urge to move them. Individuals diagnosed with PD may experience obstructive sleep apnea or other sleep disorders related to breathing. The presence of sleep disorders may result in a heightened level of daytime sleepiness, ultimately leading to a reduction in the overall quality of life of the affected individual.^[15]^ Physicians should devise suitable treatment regimens for sleep disorders in patients with PD based on the patient’s particular symptoms, disease progression, and other pertinent factors. These treatment plans may include pharmacological interventions, lifestyle adjustments, and other nonpharmacological therapies.

Insomnia is a widely observed symptom in individuals with Parkinson disease, with an estimated incidence rate of approximately 60%. Insomnia can be attributed to several significant factors, including the disease itself, motor symptoms, medications that affect the dopaminergic and anticholinergic systems, and depression. A significant correlation was observed between insomnia and both restless limb syndrome and depression as well as levodopa dosage. Sleep maintenance disorders, such as sleep fragmentation, affect a significant proportion (74%) of PD patients (88%). These disorders have been linked to motor symptoms, autonomic nervous system diseases, RLS, and respiratory disorders.^[16]^ Early awakening can be attributed to various factors such as nocturia, dystonia, and sensory disorders. The prognosis of Parkinson is adversely affected by insomnia, with sleep difficulties being the primary symptom in 10% of patients with advanced Parkinson disease. The findings of this investigation indicate that individuals with Parkinson disease experience a decrease in total sleep time, sleep efficiency, and REM sleep latency, along with an increase in the duration of the first stage of sleep and a decrease in the duration of the REM sleep stage. Nonetheless, no significant difference was observed in the frequency of awakening. The present investigation revealed that STN-DBS intervention resulted in amelioration of both sleep onset difficulties and sleep maintenance disorders, as measured by the PDSS. The amelioration of insomnia is influenced by various factors, while alterations in motor symptoms and dopamine dosage have a favorable impact on sleep.

REM, Rapid Eye Movement Sleep Behavior Disorder, and dream enactment behavior often present atypical sleep-related manifestations. The prevalence of REM sleep behavior disorder RBD among individuals diagnosed with PD ranges from 25% to 50%. The precise cause of RBD remains elusive; however, it has been hypothesized to be associated with the functioning of the brainstem nucleus. Approximately 30% of patients who use levodopa for an extended period of time encounter intense hallucinations linked to dopamine dosage during the night, and mitigating the symptoms can be achieved by decreasing nocturnal consumption. RBD has been observed to exhibit a positive correlation with increased levels of exercise intensity and nonathletic performance. The lack of a control group precluded the exclusion of the possibility of levodopa reduction in patients who experienced postoperative benefits from DBS and whose nocturnal mental symptoms were alleviated due to surgical intervention.

Sleep disorders encompass a variety of conditions, including sleep-related motor disorders such as RLS and sleep respiration disorders. The application of continuous positive pressure ventilation has been found to mitigate the manifestations of sleep apnea, a condition associated with both airway disease and brainstem impairment. Nocturia in patients with PD has been observed to cause sleep disturbances. Additionally, Parkinson has been identified as a risk factor for nocturia in elderly males. The efficacy of DBS in significantly improving nocturia remains inconclusive; however, some studies have indicated its potential to alter bladder activity. The absence of explicit directives for the treatment of nocturia in individuals with Parkinson disease necessitates reliance on overarching principles applicable to geriatric patients. The impact of DBS on RLS remains uncertain; however, certain studies have proposed that it could potentially yield advantageous outcomes. The potential biological association between PD and RLS has been a topic of debate among researchers, as evidenced in the existing literature. Pulmonary dysfunction is considered a plausible risk factor for sleep-disordered breathing in individuals with PD. However, the root cause may be analogous to that observed in a broader population.

According to research, STN-DBS has the potential to improve sleep quality in individuals with Parkinson disease, specifically in terms of sleep duration, sleep efficiency, and restless legs syndrome.^[8]^ Nevertheless, the amelioration of fragmented sleep was deemed to be unsatisfactory. Several studies have reported that stimulation of the STN in patients with PD affects the duration of both slow-wave sleep and REM sleep, as observed through polysomnography. After discontinuation of the stimulus, sleep returned to its initial state, suggesting that STN stimulation can modulate central function. However, some studies have suggested that patients with PD who undergo DBS may experience persistent and potentially exacerbated symptoms of REM sleep behavior disorders and periodic limb movements.

The decrease in dopaminergic medications after DBS has a notable impact on the sleep patterns of individuals with PD.^[17]^ DBS improves sleep quality and reduces excessive somnolence in patients with PD. However, it remains unclear whether this improvement is attributable to dopaminergic medications administered post-surgery.^[18]^ Furthermore, postoperative complications associated with DBS may affect the quality of sleep experienced by patients. The prevalent complications associated with DBS are: Surgical complications such as intracerebral hemorrhage, venous thrombosis, postoperative epilepsy, postoperative altered state of consciousness, and inadequate electrode placement have been reported in the literature. Implants may lead to various complications, including, but not limited to, electrode displacement, skin perforation leading to hardware leakage, knife edge infection, and electrode wire fracture. The complications associated with nerve stimulation include dysarthria, dysphagia, sensory aberrations, nausea, disorientation, and diaphoresis. To achieve optimal therapeutic outcomes for individuals with Parkinson, it is imperative to have a proficient surgical team, accurate electrode placement, minimized postoperative complications, and personalized stimulation management.

Methodical evaluation and sleep control are of paramount importance for individuals with Parkinson. Sleep disorders are commonly diagnosed using various tools, such as polysomnography, Epworth sleepiness scale, Pittsburgh sleep quality index, and PDSS.^[18,19]^ The PDSS is a specialized 15-item visual analog scale designed specifically for individuals with Parkinson disease, among the various instruments available. The present investigation has shown that DBS of the STN enhances the PDSS score at our institution, corroborating the outcomes of previous studies.

The present investigation, which centered on STN-DBS, did not consider Tolleson et al^[11]^ 2016 GPi study owing to the absence of statistical significance. Instead, it builds upon Lukas et al 2016 systematic review by integrating original data and measuring pooled outcomes. The findings demonstrated a noteworthy enhancement in sleep quality, with a pooled mean difference of 20.41 and a 95% confidence interval of 13.03 to 27.79. In contrast to the study conducted by Lukas et al,^[20]^ we employed a more rigorous methodology to verify the adequacy of our sample size and the soundness of our findings. While Lukas et al reported an improvement in PDSS scores, their study did not employ trial sequential analysis or other robust statistical approaches to assess the sufficiency of the sample size or the stability of the outcomes. Our study addresses these limitations by incorporating a comprehensive evaluation of heterogeneity and a more stringent statistical analysis, providing more robust and reliable evidence for the positive impact of STN-DBS on sleep quality in patients with PD. However, it is imperative to conduct additional RCTs to investigate the PDSS subscores and the influence of diverse targets and anesthesia protocols on non-motor disorders. The present investigation corroborates the findings of Kleiner–Fisman et al^[21]^ meta-analysis conducted in 2006, which reported a mean decrease of 55.9% in L-dopa equivalents following surgical intervention. This underscores the significance of exploring non-motor impairments that affect the well-being of patients.

Our study’s limited sample size and variability may affect reliability and generalizability. Due to our small sample size, chance fluctuations may affect our results, limiting their application to a larger patient population. Study design, patient demographics, assessment methods, and monitoring periods may cause outcome heterogeneity. This may complicate our findings and limit their usefulness. We also understand that using subjective sleep assessment methods like the PDSS instead of objective ones like polysomnography may affect our results. Polysomnography can eliminate patients’ biases and perceptions from sleep assessments.

First, increase the research sample size. Extensive RCTs may benefit prospective studies. Considering effect magnitude, occurrence rates, and statistical methodologies, we recommend early investigation on several hundred people. Thus, a control group is needed to compare deep brain stimulation to other Parkinson disease treatments or no treatment. Future studies should use consistent assessment methods, monitoring durations, and diversity-related variables. To reduce selection bias and standardize baseline variables including age, gender, and disease stage, future research should use random assignment. Future study should document and disclose all intervention methods and significant investigative procedures to find heterogeneity factors through subgroup analysis or meta-regression.

## 5. Conclusion

In brief, the results of this systematic review and meta-analysis indicate that deep brain stimulation of the subthalamic nucleus can improve sleep quality among individuals diagnosed with Parkinson. Furthermore, there was a significant reduction in postoperative Unified Parkinson Disease Rating Scale-III scores and medication use in individuals with Parkinson. The adequacy of the sample size allowed for conclusive inferences, thereby obviating the need for additional self-controlled experiments.

## Acknowledgements

This research was supported by the Medical and Health Science and Technology Plan Project of Zhejiang Province (Project No2023KY549). We appreciate the technical support provided by the clinical research personnel and students of our laboratory.

## Author contributions

**Conceptualization:** Keying Zhu, Zhonglei Lu.

**Data curation:** Keying Zhu, Zhonglei Lu.

**Formal analysis:** Yulun Wu, Zhonglei Lu.

**Investigation:** Sun Peng, Yulun Wu, Yuanyuan Zhao, Zhonglei Lu.

**Methodology:** Sun Peng, Yuanyuan Zhao.
